# Successful Surgical Management of Optic Disc Pit Maculopathy Without Internal Membrane Peeling

**DOI:** 10.4103/0974-9233.65495

**Published:** 2010

**Authors:** Fahad Diab, Khaled Al-Sabah, Abdullah Al-Mujaini

**Affiliations:** Department of Ophthalmology, Al-Bahar Ophthalmology Centre, Kuwait City, Kuwait; 1 Department of Ophthalmology, Sultan Qaboos University, Muscat, Sultanate of Oman

**Keywords:** Optical coherence tomography, Optic disc pit, Serous macular detachment

## Abstract

Optic disc pit is an excavation of the optic nerve head usually seen in association with other abnormalities of the optic nerve, peripapillary retina, or posterior vitreous detachment. In 50% the cases, it might be associated with serous macular detachment. The prevailing theory that explains this disorder is that subretinal fluid which is derived from liquefied vitreous passes through the optic disc pit and elevates the macula. In this study, we report a case of serous macular detachment complicating optic disc pit in a young male patient treated surgically without internal limiting membrane peeling and showed dramatic improvement of vision after 1 year.

## INTRODUCTION

Optic disc pit is a rare finding that occurs in <0.01% of ophthalmology patients.^1^ It may be associated with a large optic nerve head, inferior colobomata of the optic disc, and inferonasal retinal colobomata supporting the hypothesis that the optic disc pit develops from incomplete closure of the inferior of the embryonic fissure.

An optic pit appears as a gray, yellow, or black oval excavation most often on the temporal side of an enlarged optic disc. It may occur centrally in about 20%. Up to two-thirds of patients with optic disc pits have blurred vision because of serous macular detachment that starts at the edge of the pit and may extend to the fovea.^2^ With chronic serous detachment, precipitates may occur on the posterior surface of the retina, and chronic cystoid changes appear in the fovea. The source of the subretinal fluid is controversial, and it is suggested that the detachment may involve the passage of vitreous fluid from the area of the pit to the subretinal space. Other sources of fluid may be abnormal blood vessels at the base of the pit or subarachnoid space.^3^ Recent publications have suggested that both posterior vitreous detachment and macular traction play a vital role in the pathogenesis of optic disc pit maculopathy.^4^

We report a case of serous macular detachment complicating optic disc pit in a young male patient treated surgically without internal limiting membrane (ILM) peeling and show dramatic improvement of vision after 1 year.

## CASE REPORT

A 36-year-old man presented with a history of painless, progressive reduction of central vision of the left eye of 1-month duration. There was no history of trauma. His medical history was not significant.

Ophthalmic examination showed a best-corrected visual acuity (BCVA) of 20/20 and CF 2 m in his right and left eyes, respectively. Both pupils were reacting normally to light, and intraocular pressure was normal. Slit lamp biomicroscopy was unremarkable on both eyes.

**Figure 1:**
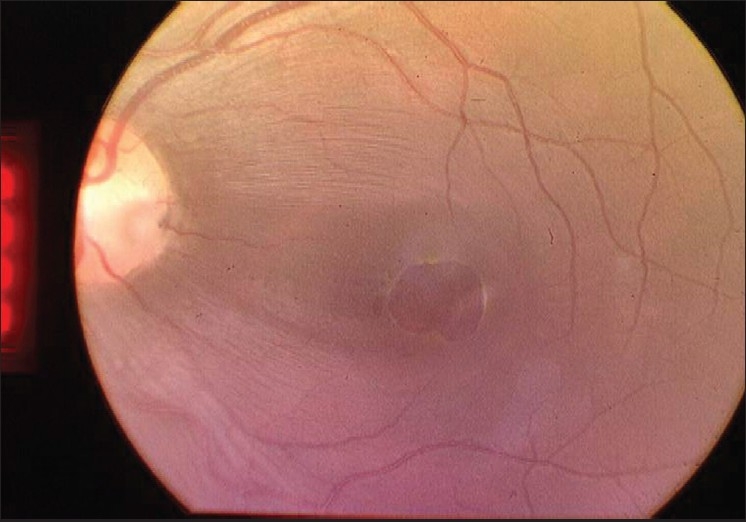
Fundus photo for the left eye on presentation showing temporal optic disc pit and serous macular detachment

Dilated fundus examination of the left eye revealed oval, grayish-yellowish crater-like depression on the temporal aspect of the optic disc suggestive of optic pit along with serous macular detachment measuring about 1.5 DD [Figure 1]. There was a thinning of the retinal layers giving the impression of a pseudo macular hole.

**Figure 2:**
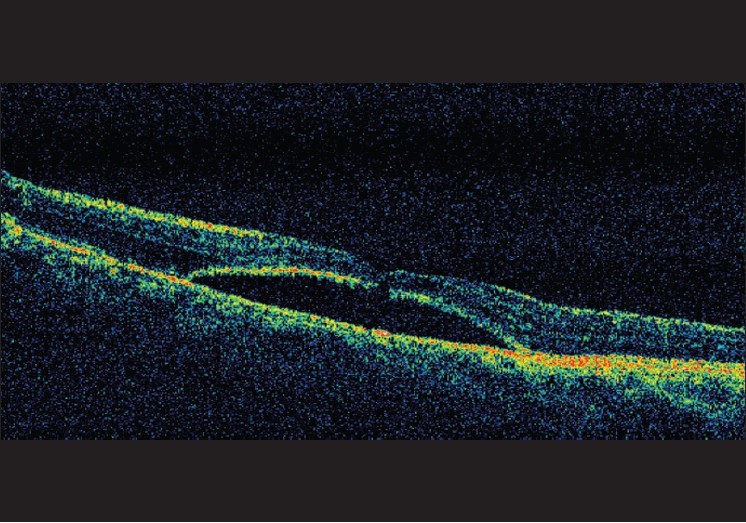
OCT for the same eye on presentation showing elevation and thickening of the macula

Additionally, optical coherence tomography (OCT) with retinal map analysis of the left eye showed serous macular detachment with central thickness measuring 708 *µ*m (normal 150–250 *µ*m) and total volume of 11.68 cumm (normal 6–7 cumm) [Figure 2].

The patient underwent standardized three ports pars plana vitrectomy together with air fluid exchange using 10% C3F8 and argon endo-laser temporal to the disc obtaining grade 1 laser burns in two to three rows. The ILM was visualized during the procedure, but was left in place to test the outcome of managing the optic disc pit maculopathy without peeling the membrane.

**Figure 3:**
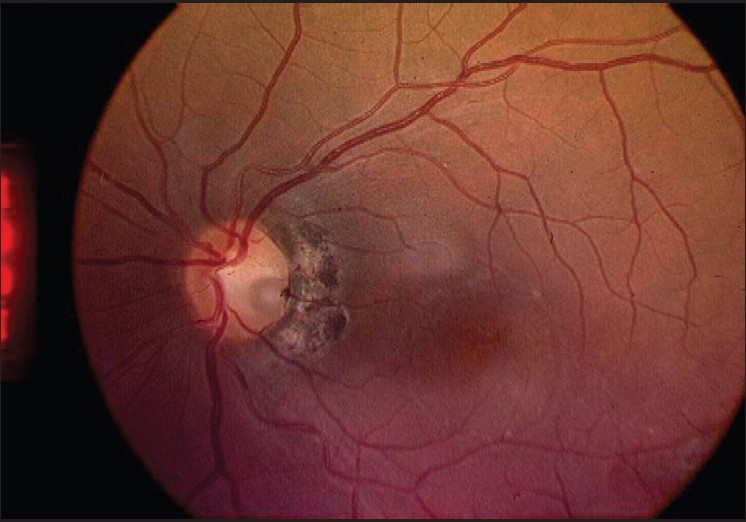
Fundus photo taken one year after the surgery showing photocoagulation scar temporal to the disc

**Figure 4:**
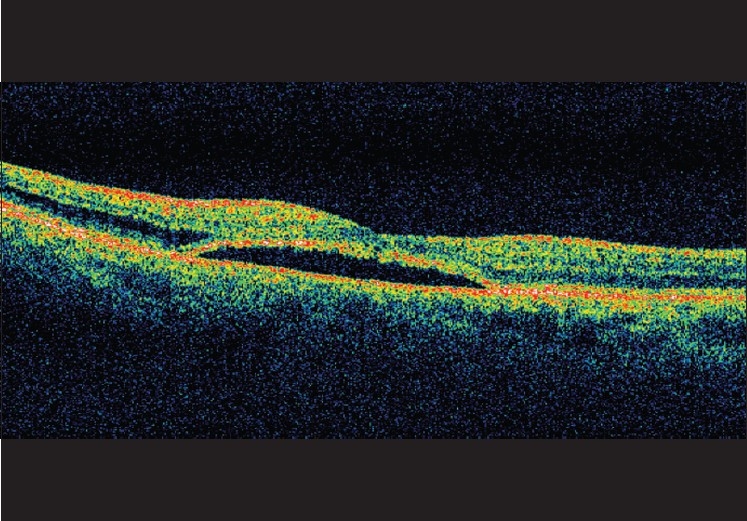
OCT taken 1 year after the surgery showing reduction of the subretinal fluid

Three months after the surgery, the patient’s BCVA was 20/100 and retinal examination showed reduction in the subretinal fluid documented both clinically and by OCT (central macular thickness of 474 *µ*m with total volume of 9.73 cumm). At 1 year, his BCVA improved further to 20/40 with minimum subretinal fluid demonstrated by fundus examination [Figure 3] and OCT (central macular thickness measuring 281 *µ*m with total volume of 7.94 cumm) [Figure 4].

## DISCUSSION

A large temporal optic disc pit is most often associated with serous sensory retinal detachment. The source of the subretinal fluid is controversial. In one report, scanning laser ophthalmoscopy revealed a cyst-like structure terminating at the pit in the premacular vitreous.^5^ During ocular movement, this structure moved vigorously and seemed to exert traction on the pit. This traction on the pit may be a significant factor in the development of serous macular detachment in these patients.^6^ A tear in the diaphanous tissue overlying the optic disc pit is responsible for the development of serous macular detachment and is consistent with findings in similar conditions, such as retinal detachment in association with chorioretinal coloboma.

Theoretically, an ideal procedure for reattaching the macula associated with an optic disc pit would be the one that reroutes the flow of fluid from the pit. If the fluid is of cerebrospinal origin, then an optic nerve sheath fenestration might divert the flow to the orbit.

A number of treatment options have been reported to treat serous retinal detachments that may occur in combination with optic disc pits which include laser photocoagulation with or without intravitreal tamponade using gas or silicone oil.^7^ Several cases of resolution of subretinal fluid after photocoagulation have been reported, but the resolution might take upto 6 months.^8^ Possible side effects of laser photocoagulation near the disc include paracentral scotomas, nonimprovement of visual acuity, and low success rate in resolution of serous macular detachment.

Tractional forces could explain the delay of macular detachment in young adulthood and the frequency of treatment failure after laser photocoagulation and gas tamponade. Vitrectomy with ILM peeling and gas tamponade without any additional laser photocoagulation seems to be sufficient for the treatment of optic disc pit maculopathy.^4^,^7^ Recently, Jalil *et al*. has described a new surgical approach of draining subretinal fluid in optic disc pit maculopathy using 42-gauge cannula.^9^

In our case, we decided not to attempt ILM peeling because the treatment of optic disc maculopathy, as mentioned earlier, remains controversial. However, because of the relatively poor visual prognosis, treatment should include the formation of a barricade to fluid movement as well as sealing and relief of traction from the hole. The technique of closing the defect with vitrectomy, gas and laser without ILM peeling has not been explored widely. We aimed to test this modality in this patient and found that it was effective in resolving the macular detachment.

In summary, we report a case of serous macular detachment complicating optic disc pit in a young male patient using standard vitrectomy without ILM peeling that showed dramatic functional and anatomical improvement at 1 year follow-up. Further studies are required to evaluate this technique, although the implementation of large-series studies remains a challenge because of the rarity of cases of optic disc pit maculopathy.
